# Gelatinous drop-like amyloid in *FOXC2* distichiasis syndrome: a case report

**DOI:** 10.1186/s12886-026-04800-1

**Published:** 2026-04-11

**Authors:** Gabriel Sexton, Nadine H. Oury, Kelly Schooping Tripi, Ken K. Nischal, Charleen T. Chu

**Affiliations:** 1 Department of Pathology, Division of Neuropathology, University of Pittsburgh School of Medicine, Pittsburgh, PA USA; 2 Medical Scientist Training Program and Pittsburgh Center for Pain Research, University of Pittsburgh, Pittsburgh, PA USA; 3 Vision Institute, University of Pittsburgh Medical Center, Pittsburgh, PA USA; 4 Division of Pediatric Ophthalmology, Strabismus and Adult Motility, UPMC Children’s Hospital of Pittsburgh, Pittsburgh, PA USA; 5 Department of Ophthalmology, University of Pittsburgh School of Medicine, Pittsburgh, PA USA

**Keywords:** Gelatinous drop-like dystrophy, Corneal amyloid, Distichiasis, *TACSTD2*, *FOXC2*, Case report

## Abstract

**Background:**

Gelatinous drop-like dystrophy is a rare autosomal recessive disorder caused by pathogenic variants in the *TACSTD2* gene and characterized by subepithelial amyloid deposits in the cornea. Alterations in *FOXC2* are seen in lymphedema-distichiasis syndrome, in which patients classically exhibit lymphedema of the extremities and a double row of eyelashes. This report links alterations in the *FOXC2* gene to a corneal phenotype that is histologically identical to that seen in gelatinous drop-like dystrophy.

**Case presentation:**

A 14-year-old male with congenital distichiasis presented with chronic photosensitivity and progressively worsening vision over several years. On examination, gelatinous-appearing lesions were observed on the inferior third of each cornea with neovascularization. Genetic testing identified a likely pathogenic alteration in *FOXC2*, a gene linked to lymphedema-distichiasis syndrome. No genetic alterations of corneal dystrophy were identified. The patient underwent excisions of the corneal lesions with simple limbal epithelial transplant, amniotic membrane transplant and temporary central tarsorrhaphy. The pathologic features of the corneal lesions resembled gelatinous drop-like dystrophy. One side was free of inflammation, while the other side showed a granulomatous response to the amyloid, which is unusual in this site. Potential pathogenic and clearance mechanisms are discussed. There has been no recurrence of the corneal lesions in approximately 3 years of follow-up.

**Conclusions:**

This case presents a new link between *FOXC2* alterations and a histological phenotype that resembles gelatinous drop-like dystrophy (GDLD-like), emphasizing the importance of clinical, pathologic, and molecular correlation. Granulomatous responses to amyloid are unusual in this immune privileged site.

## Background

Gelatinous drop-like dystrophy (GDLD) is a rare autosomal recessive disorder caused by pathogenic bi-allelic variants in the *TACSTD2* gene and characterized by subepithelial amyloid deposits across the whole cornea. Ultrastructural studies demonstrate loosening of cell-cell junctions in the corneal epithelium. TACSTD2 is a cell adhesion molecule, and leakage of tear and serum components including lactoferrin through dysfunctional epithelial cell junctions is thought to contribute to amyloid formation (3). Affected patients experience diminished visual acuity and photophobia within the first decade of life. Over time, the amyloid deposits develop into raised, gelatinous lesions across the whole cornea. Treatment usually consists of laser photoablation or corneal scraping [1]. Corneal transplantation may also improve vision in severe cases but is often followed by recurrent amyloid [2]. Few cases of GDLD have been reported outside of Japan, where its incidence is relatively increased [1–3]. 

Pathogenic variants in *FOXC2* are primarily responsible for lymphedema-distichiasis syndrome (LDS), a rare autosomal dominant disorder characterized by double rows of eyelashes and lymphedema affecting the limbs [4]. FOXC2 is a transcription factor expressed in the developing mesodermal mesenchyme of the head, kidney, and skeleton. The LDS phenotype is variable and may include cardiac, skeletal, and other developmental abnormalities.

The following case demonstrates a GDLD-like phenotype in a patient with confirmed *FOXC2*-linked distichiasis, implicating a new gene in the pathogenesis of corneal amyloid deposition. We discuss possible pathogenic mechanisms and demonstrate successful surgical management without recurrence.

## Case presentation

A 14-year-old male with congenital distichiasis presented with chronic photosensitivity and several years of slowly worsening vision. His history included multiple lash electrolysis and lower lid splitting between ages 4–7 with cryotherapy, elsewhere. There was no family history of genetic conditions or similar symptoms. Visual acuity was 20/30 in the right eye and 20/100 in the left.

**Fig. 1:**
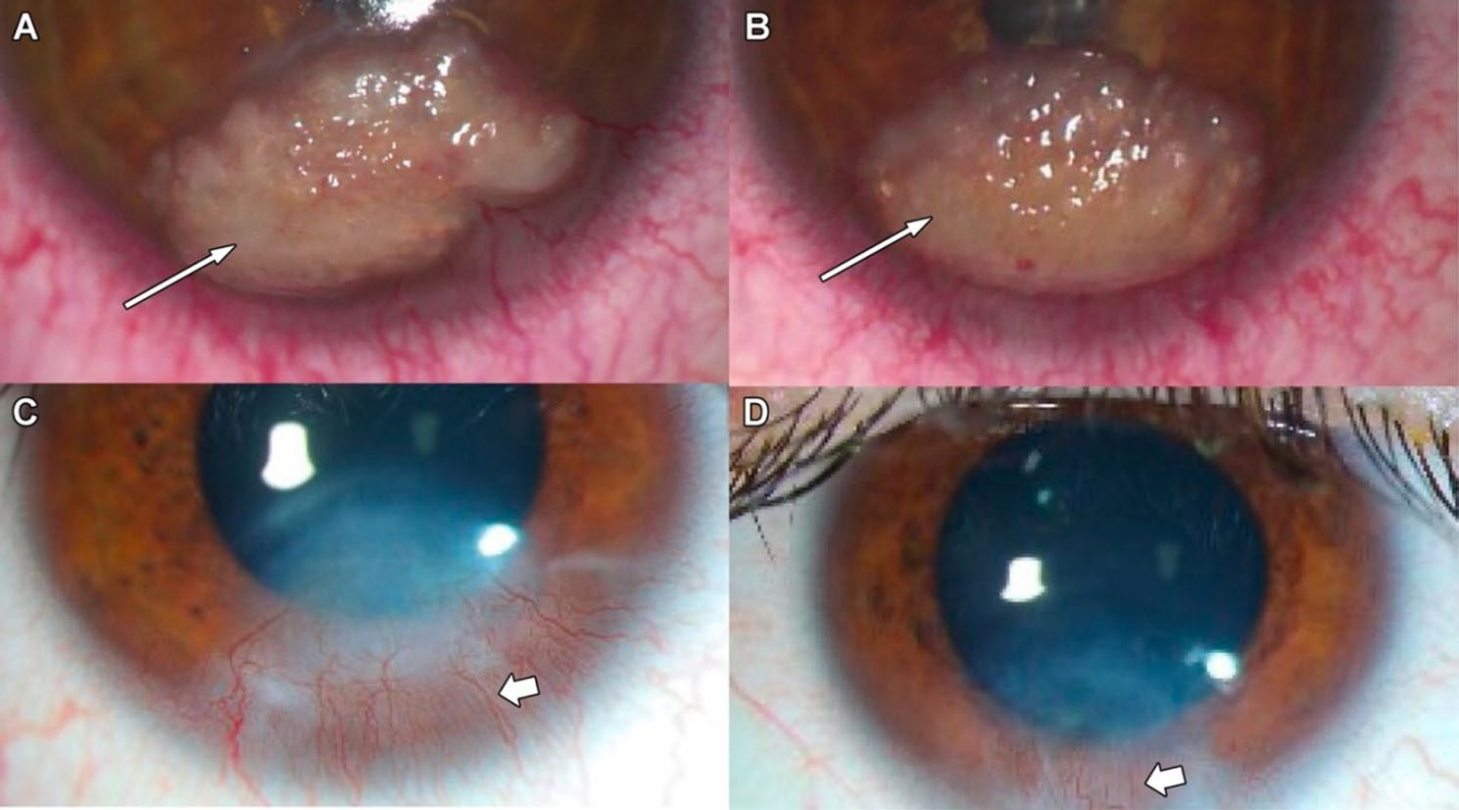
Pre- and Post-operative Appearance of the Corneas. Preoperative images of the (**A**) right and (**B**) left cornea illustrate the creamy gelatinous appearance of the lesions (thin arrows). Postoperative images of the (**C**) right and (**D**) left eye at the two-year follow-up visit show neovascularization (thick arrows) with no recurrence

A gelatinous-appearing lesion was present on each cornea (Fig. 1). The lesions were subepithelial and located to the anterior stroma by optical coherence tomography. Unlike classic gelatinous drop-like dystrophy (GDLD), which typically affects the entire cornea, the GDLD-like lesions in the current case involved the lower third of each cornea. Fundoscopy revealed a left optic nerve coloboma.

Genetic testing was performed at a CLIA-certified laboratory (Blueprint Genetics) utilizing next-generation sequencing for *FOXC2*, a gene linked to lymphedema-distichiasis syndrome (LDS), and a panel of genes related to corneal dystrophy that included *TACSTD2*. This panel targets protein coding exons, exon-intron boundaries (+/- 20 bps) and selected deep intronic variants, with the ability to detect single nucleotide variants, small insertion-deletions and single exon copy number variants or larger chromosome deletions and duplications. This revealed a heterozygous, likely pathogenic, nonsense variant in the *FOXC2* gene (c.915 C > A, p.(Tyr305*)). No genetic alterations of corneal dystrophy were identified.

**Fig. 2:**
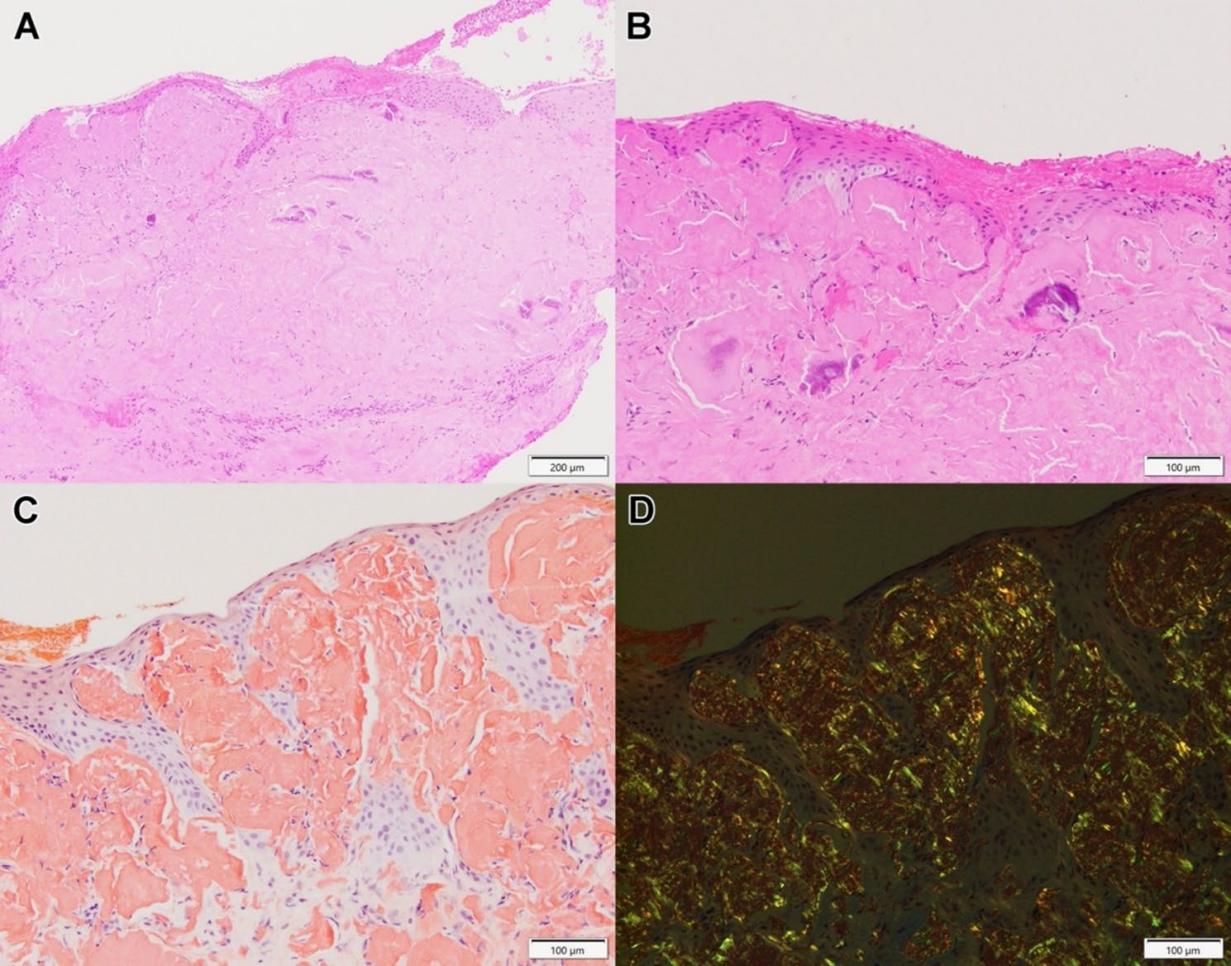
Corneal Pathology Reveals Amyloid Deposits. (**A**, **B**) Lower and higher power views of the right cornea showing extensive subepithelial deposits of amorphous eosinophilic material (hematoxylin and eosin, 100x and 200x). Focal calcifications are observed in (**B**). (**C**) The subepithelial deposits exhibit positive Congo Red staining with (**D**) yellow-green birefringence when examined under polarized light (200x). Scale bars: **A**, 200 microns; **B**-**D**, 100 microns

**Fig. 3:**
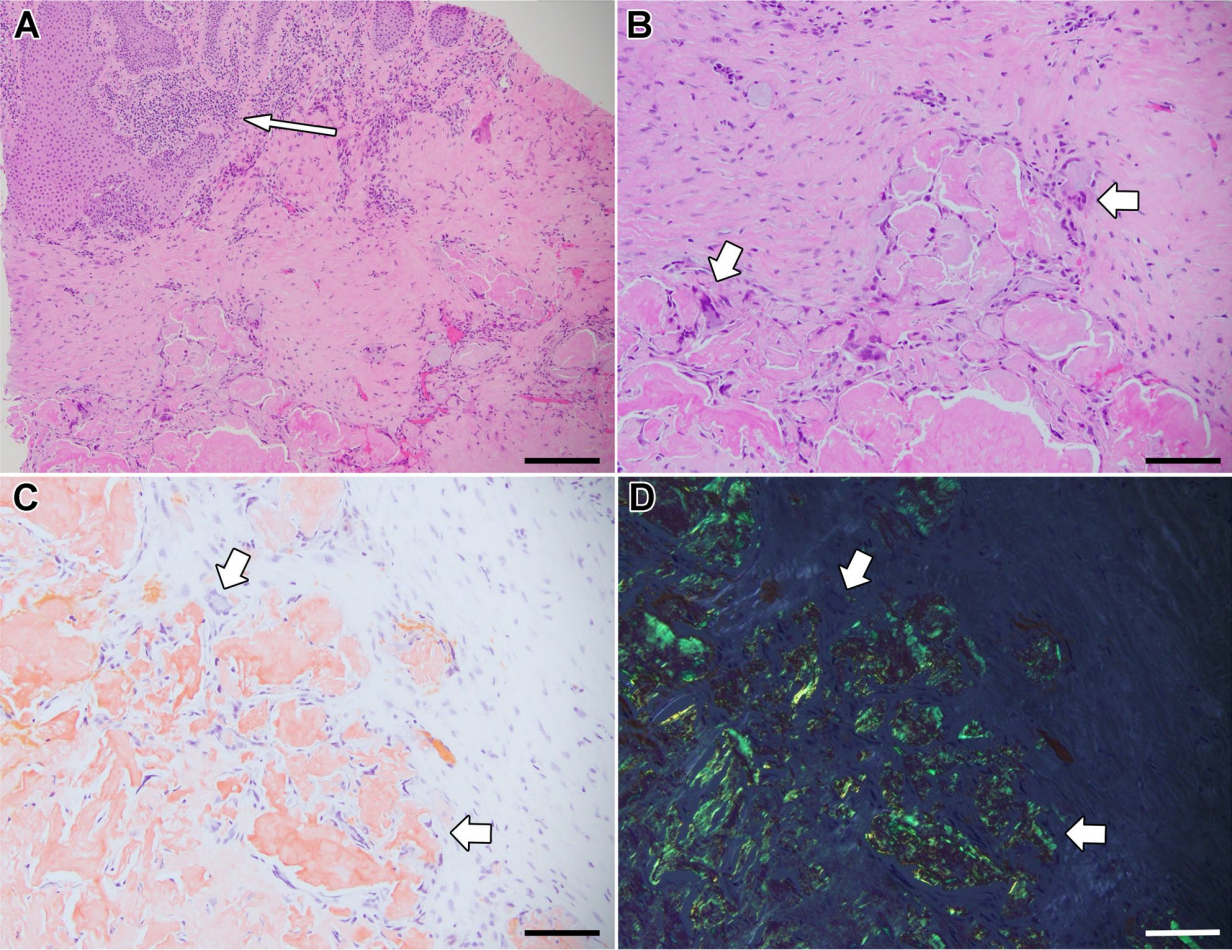
Chronic Inflammation and Giant Cell Reaction to Amyloid. (**A**) Low power view of the left cornea revealing patchy chronic inflammation superficially (thin arrow), and deeper deposits of amorphous eosinophilic material (hematoxylin and eosin, 100x). Higher power views (**B**-**D**, 200x) reveal multinucleated giant cells surrounding some of the deposits (thick arrows). (**C**) The material is positive by Congo Red staining and (**D**) shows yellow-green birefringence under polarized light (200x). In some places, the amyloid shows scalloped edges with multinucleated cells within the indented area (**C**-**D**, lower right arrow). Scale bars: **A**, 200 microns; **B**-**D**, 100 microns

The patient underwent excisions of the corneal lesions with simple limbal epithelial transplant, amniotic membrane transplant and temporary central tarsorrhaphy, a technique that has shown success in a different clinical context [5]. Histologic examination demonstrated extensive amorphous stromal deposits, confirmed as amyloid by Congo Red staining (Figs. 2 and 3), with focal microcalcifications.

There was no inflammation on the right side (Fig. 2), whereas the left cornea exhibited superficial lymphoplasmacytic inflammation (Fig. 3A). Beneath the inflamed region, amyloid deposits were limited to the mid-stroma with numerous multinucleated giant cells surrounding and sometimes scalloping the amyloid deposits (Fig. 3). In regions of the left cornea lacking inflammation (not shown), the amyloid deposits abutted the epithelium with an identical appearance to the right side. No hyaline deposits were observed by Masson trichrome staining. Overall, the pathologic features of the corneal lesions resemble GDLD.

In the first 2 years after surgery, the patient received 4 treatments of bevacizumab (625mcg) subconjunctivally in each eye to treat residual corneal neovascularization (Fig. 1C-D) with no recurrence. At 3 years after surgery, the patient’s visual acuity was 20/20 in the right eye with improvement to 20/40 in the left eye (best corrected visual acuity).

## Discussion

This case links a new gene and syndrome to the pathogenesis of GDLD-like corneal lesions. Our patient developed GDLD-like corneal lesions in the setting of distichiasis and *FOXC2* alteration.

GDLD is characterized by subepithelial amyloid deposits in the cornea caused by pathogenic variants of the tumor-associated calcium signal transducer 2 (*TACSTD2*) gene [6]. The TACSTD2 protein binds directly to claudins, namely CLDN1 and CLDN7, to stabilize them and keep tight junctions intact [3]. Dysfunctional TACSTD2 results in decreased claudin levels and nonfunctional tight junctions. The loss of this epithelial barrier in the cornea allows for lactoferrin, a major component of tears, to disperse through the barrier and form amyloid aggregates in the cornea [3]. 

In this case, there were no variations in *TACSTD2*, but instead a heterozygous, likely pathogenic, nonsense variant in the *FOXC2* gene (c.915 C > A, p.(Tyr305*)) was found. *FOXC2* encodes a regulatory transcription factor that plays a role in neural crest cell derived corneal development [7, 8] and mutations are known to be associated with distichiasis as seen in our patient [9]. Mutations in *FOXC2* have also been shown to cause corneal neovascularization,^8^ and FOXC2 is essential for maintaining lymphatic barrier function [10]. 

In humans, FOXC2 and several claudins are highly expressed in barrier-type epithelial cells including the ocular epithelium [11, 12]. In tumor cells, FOXC2 expression is often inversely correlated with claudin levels [13]. While there are no studies of FOXC2 function in this context in ocular cells, claudins play an important role in maintaining lymphatic as well as epithelial tight junction integrity [14]. Future studies addressing the potential impact of FOXC2 on claudin expression in cells relevant to lymphedema and corneal barrier function are needed.

Different patterns of amyloid deposition may also occur as secondary corneal amyloidosis (SCA), a rare and incompletely understood process in the setting of chronic corneal irritation and inflammation. Patients with SCA usually present in adulthood with amyloid deposits localized to the site of inflammation or irritation. Corneal neovascularization is often accompanied by stromal inflammation, which is known to weaken epithelial tight junctions [15, 16]. Studies of the amyloid material in SCA suggest that, like GDLD, it is composed of lactoferrin [17]. 

In the current case, chronic inflammation was observed only on one side, indicating that inflammation was not required for amyloid deposition. Nevertheless, it is possible that corneal irritation due to distichiasis elicited repeated microtrauma of the lower cornea on the background of corneal neovascularization, resulting in amyloid deposition only in the lower third of the cornea. The possibility of variant *FOXC2*-associated epithelial barrier dysfunction will require future experimental studies.

In orbital and systemic amyloidosis, a granulomatous response to amyloid is frequently reported [18, 19]. However, a foreign body giant cell response associated with corneal amyloid is extremely rare and, to our knowledge, has been previously described in only one patient in a series of GDLD cases [20]. This may be attributed to immune privilege of the cornea, as lymphocytes and multinucleated giant cells do not enter the cornea unless immune privilege is breached [21, 22]. It is possible that neovascularization of this eye was accompanied by disruption of corneal immune privilege, allowing a foreign body giant cell response. Interestingly, the multinucleated giant cells in the current case were present in scalloped indentations of the amyloid material; moreover, the immediate subepithelial region of the left cornea showed an amyloid-free fibrotic zone, compatible with inflammatory clearance.

In conclusion, this case presents a new link between *FOXC2* alterations and a histological GDLD-like phenotype, emphasizing the importance of clinical, pathologic, and molecular correlation. Inflammation is not required for amyloid deposition, but a foreign body response to corneal amyloid may rarely be seen in severe cases.

## Data Availability

All data supporting the findings of this study are available within the paper.

## Abbreviations

GDLD: Gelatinous drop-like dystrophy
LDS: Lymphedema-distichiasis syndrome
SCA: Secondary corneal amyloidosis
